# Network-Like Platinum Nanosheets Enabled by a Calorific-Effect-Induced-Fusion Strategy for Enhanced Catalytic Hydrogenation Performance

**DOI:** 10.3389/fchem.2021.818900

**Published:** 2022-01-05

**Authors:** Ting-Wen Chen, Da-Wei Pang, Jian-Xin Kang, Dong-Feng Zhang, Lin Guo

**Affiliations:** ^1^ Key Laboratory of Bio-Inspired Smart Interfacial Science and Technology, School of Chemistry, Beijing Advanced Innovation Center for Biomedical Engineering, Beihang University, Beijing, China; ^2^ School of Physics, Beihang University, Beijing, China; ^3^ Institute of Microstructure and Property of Advanced Materials, Beijing University of Technology, Beijing, China

**Keywords:** network-like, Pt nanosheets, spontaneously combustion, calorific-effect-induced-fusion, catalytic hydrogenation

## Abstract

In this paper, we report the construction of network-like platinum (Pt) nanosheets based on Pt/reduced graphite oxide (Pt/rGO) hybrids by delicately utilizing a calorific-effect-induced-fusion strategy. The tiny Pt species first catalyzed the H_2_-O_2_ combination reaction. The released heat triggered the combustion of the rGO substrate under the assistance of the Pt species catalysis, which induced the fusion of the tiny Pt species into a network-like nanosheet structure. The loading amount and dispersity of Pt on rGO are found to be crucial for the successful construction of network-like Pt nanosheets. The as-prepared products present excellent catalytic hydrogenation activity and superior stability towards unsaturated bonds such as olefins and nitrobenzene. The styrene can be completely converted into phenylethane within 60 min. The turnover frequency (TOF) value of network-like Pt nanosheets is as high as 158.14 h^−1^, which is three times higher than that of the home-made Pt nanoparticles and among the highest value of the support-free bimetallic catalysts ever reported under similar conditions. Furthermore, the well dispersibility and excellent aggregation resistance of the network-like structure endows the catalyst with excellent recyclability. The decline of conversion could be hardly identified after five times recycling experiments.

## Introduction

Platinum (Pt) plays an indispensable role in industrial production owing to its unique and versatile catalytic capability (Hao et al., 2014). Considering the high cost and rare reserve of Pt (Huang et al., 2012), a key point is to explore Pt-based catalysts with high atom utilization and superior catalytic performance (Gong et al., 2014; Song et al., 2017). Up to now, the commonly adopted strategies include alloying Pt with non-precious metals (such as Fe, Co, Ni, etc.) (Yang et al., 2017; Zeng et al., 2017; Li et al., 2021), modulating the exposed surface crystallography (to get specific facet or high-index facet) (Cao et al., 2017; Wang et al., 2017; Dong et al., 2020) and/or constructing unique morphologies (such as core shell, framework, hollow, etc.) (Ge et al., 2018; Lin et al., 2018; Liu et al., 2021).

Owing to the high surface energy aroused by the size effect, nanoparticles inevitably suffer from surface ripening and agglomeration (Chung et al., 2018), which could lead to the deterioration of catalytic activity (Pasricha et al., 2009). Supporting substrates are often used to improve the dispersibility of the nanocatalysts (Tong et al., 2017). Furthermore, the metal-support interaction might also facilitate the catalytic performance optimization (Jackson et al., 2017; Meng et al., 2017). However, like a coin has two sides, the supported catalysts also have adverse effects. 1) The corrosion of the supporting substrates in the reaction media restrains their application. For example, metallic oxides, a kind of widely used carrier, are unstable in acidic condition. Similarly, carbon-based substrates are found easily corroded during electro-catalysis process (Jiang et al., 2016a). 2) Additional components might deteriorate the catalytic performance owing to the unpredicted metal-support interaction though the catalyst could be stably immobilized on the substrate (Jayakumar et al., 2017). 3) The substrates could increase the diffusion length and thus block the efficient accessing of the reactant to the active sites of the catalyst. 4) The increased total mass or volume with the introduction of supporting substrates may lead to the undesired dispersibility in the reaction media.

One core resolution is to engineer the nanocatalysts into self-supported nanoporous networks (usually called nanofoams or porous frameworks). The porous structure could not only increase the surface area but also improve the resistance to agglomeration. Furthermore, the interconnected network-like structure could accelerate the mass transfer and electron mobility (Xu and Zhang, 2014). The superior catalytic performance endowed by the featuring structure aroused great research interest on the nanofoams (Zhu et al., 2021). Strategies like templating (Li et al., 2013; Tamaki et al., 2015; Jang et al., 2020) and selective dissolution (Liu et al., 2009) have been successfully employed for the construction of Pt nanofoams. The advance up till now mainly focused on the 3D porous Pt structure, such as porous nanospheres (Li et al., 2015; Jiang et al., 2016a; Jiang et al., 2016b), periodic porous structures (Stein et al., 2008; Kim et al., 2013; Xu and Zhang, 2014), etc. Contrastively, 2D porous structures possess more extended surface, superior flexibility, and thinner pore channel, thus meriting more active sites, faster mass/electron transfer rate, better dispersibility, and stability. However, the challenge on fabrication of 2D porous Pt nanostructures greatly restricts the exploration of their applications.

Herein, we report a calorific-effect-induced-fusion strategy to construct network-structured Pt porous nanosheets (denoted as Pt PNSs thereafter). The highly dispersed Pt species catalyzed the combustion of the reduced graphene oxide (rGO) substrate, which served both as energy supplier and sacrificial structure guide reagent for the fusion of Pt cluster into network-structure. The as-prepared Pt PNSs exhibit dramatic hydrogenation catalytic ability towards unsaturated bond such as olefins and nitrobenzene.

## Materials and Methods

### Chemical Reagents and Materials

Graphite powder was purchased from Alfa Aesar. Potassium permanganate (KMnO_4_), potassium persulfate (K_2_S_2_O_8_), and phosphorus pentoxide (P_2_O_5_) were bought from Sinopharm Chemical Reagent Beijing Co., Ltd. Hydrochloric acid (HCl), sulfuric acid (H_2_SO_4_), hydrogen peroxide (H_2_O_2_), ammonium hydroxide (NH₃·H₂O), and alcohol were purchased from Beijing Chemical Reagent Company. Chloroplatinic acid (H_2_PtCl_6_·6H_2_O) was purchased from Shenyang Jinke Chemical Reagent Company. Mesitylene, styrene, and the analogue were bought from Beijing InnoChem Science and Technology Co., Ltd. All materials were used as obtained without any purification.

### Synthesis of Graphite Oxide

Graphite oxide (GO) was prepared using a modified Hummer's method. Typically, for pre-oxidation treatment, 3.0 g graphite powder was added to 15 ml concentrated H_2_SO_4_ containing 2.5 g K_2_S_2_O_8_ and 2.5 g P_2_O_5_. After stirring vigorously under 80°C for 6 h, the pre-oxidized graphite powder was collected and dried overnight. Subsequently, the powder was dispersed into 120 ml concentrated H_2_SO_4_ in an ice bath for further oxidation. Keeping the temperature no higher than 20°C, 15.0 g KMnO_4_ was cautiously added into the suspension. After stirring for about 2 h under 35°C, 250 ml deionized water was dropped in while the temperature was kept below 50°C. After stirring for 2 h, 700 ml deionized water and 20 ml H_2_O_2_ were then added into the above solution. The yellow precipitate was obtained by centrifugation and washed with 1.5 L of 2 M HCl followed by 1.5 L deionized water. The purified GO was re-dispersed in 500 ml deionized water for later use. The concentration of GO was about 5.0 mg ml^−1^.

### Synthesis of Pt PNPs

Before synthesizing the Pt PNPs, the as-prepared GO was reduced into rGO by NaBH_4_. GO solution (1 ml) was dissolved in 20 ml deionized water containing 40.0 mg NaBH_4_. The mixture was transferred into 50 ml autoclave and kept at 140°C for 6 h. After cooling down to room temperature, the rGO precipitate was collected by centrifugal separation and with ethanol and deionized water 3 times. Finally, rGO powder was obtained by freeze-drying for later use.

rGO (5.0 mg), 1 ml NH_3_·H_2_O, and 0.16 mM H_2_PtCl_6_ were dispersed in 30 ml deionized water. After stirring in 60°C water bath for 1 h, 10 ml of ascorbic acid (Vc) aqueous solution (10 mg ml^−1^) was injected into the mixture. Then, the above solution was transferred into 90°C oil bath and kept stirring for 24 h. After natural cooling to room temperature, the black precipitate was collected by centrifugation and then washed with ethanol and deionized water 3 times. The as-prepared Pt/rGO product was collected by freeze-drying. After annealing in 5% H_2_/Ar at 200°C for 2 h, Pt/rGO hybrids spontaneously burned as soon as it was exposed to air, which produced Pt PNSs.

### Catalytic Activity Measurement

Typically, 3.0 mg catalyst and 1.7 mM styrene (or p-chlorostyrene/p-methylstyrene/cyclohexene/nitrobenzene) were added into 5 ml ethanol in a 25 ml flask. Mesitylene (1.4 mM) was added simultaneously serving as internal standard substance. Before measurement, H_2_ gas was bubbled for several minutes to remove the dissolved air. With forceful stirring, the hydrogenation reaction of olefins was carried out under room temperature and normal pressure with a H_2_ balloon as the H_2_ supplier. Liquid samples were analyzed by gas chromatography (GC) equipped with a flame ionization detector and an HP-CP8713 capillary column.

The durability was assessed by using the re-used catalyst to initiate a new cycle. Specifically, as one round of the catalytic reaction was completed, the catalyst was separated from the reaction solution by centrifugation. After washing with ethanol, the recollected catalyst was dispersed into a fresh reaction solution containing the same amount of reactant reagent as the initial round for the next cycle of activity measurement.

## Results and Discussion

Calorific effect is one of the essential features of chemical reactions, which has been widely applied in our life such as giving us warmth, providing us energy, etc. It can also be exploited to compensate the energy consumption and thus relax the reaction condition or even enable the thermodynamically impossible reaction to occur by introducing extra reagents into the system according to Hess's law. A typical case is aluminothermy, a process producing great heat by oxidizing finely divided aluminum with oxygen to launch the reduction of metal oxide. Graphene is known as 2D material featuring remarkable flexibility, incredible strength, and excellent conductivity. Easy functionalization and simple removal procedure also make the graphene a novel template. Furthermore, the combustion of graphene is exothermic. It is reasonable to infer that if tiny Pt species were uniformly dispersed onto the graphene substrate, the combustion of the graphene would induce the fusion of the Pt species, and 2D network structures might be formed under the guidance of the graphene. It is not an easy task to uniformly disperse pre-formed Pt nanoparticles onto the substrate with desired density. So we adopted the *in situ* reduction strategy to load tiny Pt species. That is, Pt (II) ions were firstly uniformly adsorbed onto rGO and the thereafter *in situ* reduction generated rGO-supported Pt metallic species.

Considering that the rGO preferred to absorb cations, the neutral NH_3_ was used to substitute the negative ligand of Cl^−^ in the PtCl_6_^2−^ precursor through ligand exchange process. Typically, the mixture containing PtCl_6_^2−^ and NH_3_ was stirred at 60°C for 1 h. The color of the solution turned from yellow into colorless, a distinct character of the successful ligand exchange. Subsequently, the given amount of rGO and ascorbic acid (Vc) were introduced into the above mixture and aged at 90°C for uniform adsorption of Pt (NH_3_)_4_^2+^ onto the rGO. The achieved black precipitate was collected and characterized to give the detailed information of the transformation. The atomic ratio of Pt and N was determined as 1:4.2, approaching the theoretical stoichiometry of Pt (NH_3_)_4_^2+^ (Supplementary Figure S1). Moreover, the Pt 4f_2/7_ X-ray photoelectron spectroscopy (XPS) spectrum was dominated by peaks ascribed to Pt^2+^ locating at 73.35 eV (Supplementary Figure S1), confirming that most of the PtCl_6_^2−^ ions had transformed into Pt (NH_3_)_4_^2+^ (Cheng et al., 2020). The weak Pt^4+^-related diffractions were believed to originate from the small amount of the remaining PtCl_6_^2−^ (Kim et al., 2006). Metallic Pt-related XPS (Supplementary Figure S1) and X-ray diffraction (XRD) (Supplementary Figure S2) indicated no Pt nanoparticles formed at this stage. The high-angle annular dark field-scanning transmission electron microscopy (HAADF-STEM) images (Supplementary Figure S3) show that the Pt (NH_3_)_4_^2+^ ions were uniformly dispersed on rGO, and no nanoscaled aggregates was identified. The inductively coupled plasma atomic emission spectrometry (ICP-AES) data indicates the loading amount of Pt on rGO reached as high as 26.46 wt%.

**FIGURE 1:**
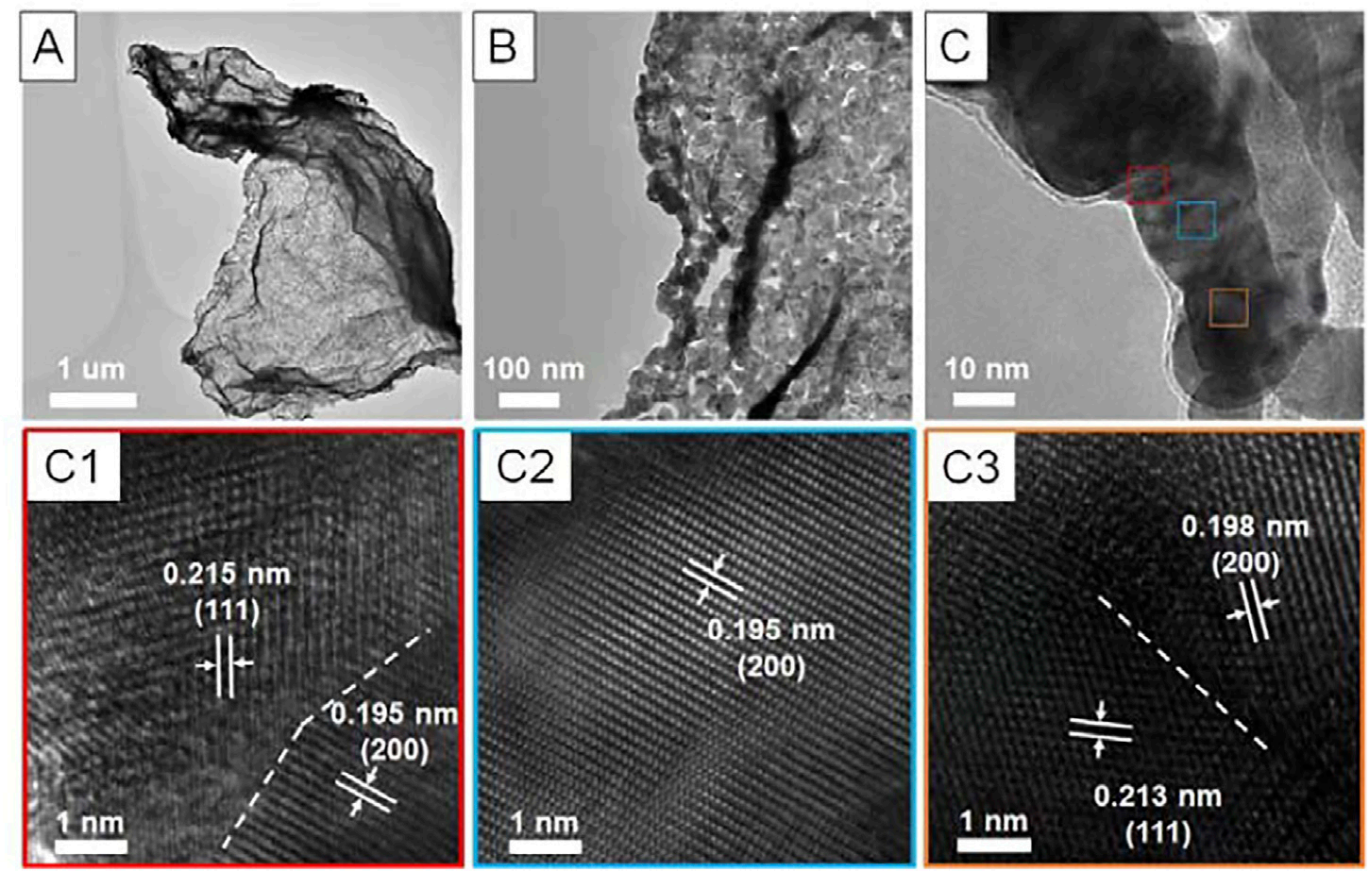
Morphology and structure characterization of the Pt PNSs. **(A–C)** different-magnified TEM images, **(C1–C3)** high-resolution TEM images recorded from the different framed regions in **(C)**. The grain boundaries were marked out by white dash line in **C1** and **C3**.

Then, the Pt (NH_3_)_4_^2+^/rGO hybrids were annealed in H_2_/Ar at 200°C for 2 h to reduce the absorbed Pt (NH_3_)_4_^2+^ into metallic Pt. Interestingly, once exposed to the air, the Pt/rGO would spontaneously burn. The transmission electron microscopy (TEM) observation (Figure 1A) reveals that the overview morphology of the product after combustion highly resembled that of rGO with 2D nanosheet structure of several micrometers in diameter. Closer observation (Figures 1B, C) clearly demonstrates the network-like nanosheet structure feature. The ligaments of the network were made of fused nanoparticles with mean diameter of about 20 nm (Figures 1B, C). The high-resolution TEM (HRTEM) characterizations (Figures 1C1–C3) show that the lattice fringe at different regions along the ligament all agree well with the (111) and (200) facet of face-centered cubic Pt (JCPDS No. 04-0802). Obviously, there were distinct grain boundaries at the junctions of adjacent nanoparticles (Figures 1C1, C3). No rGO could be further identified, indicating the complete oxidation of the rGO by combustion.

**FIGURE 2:**
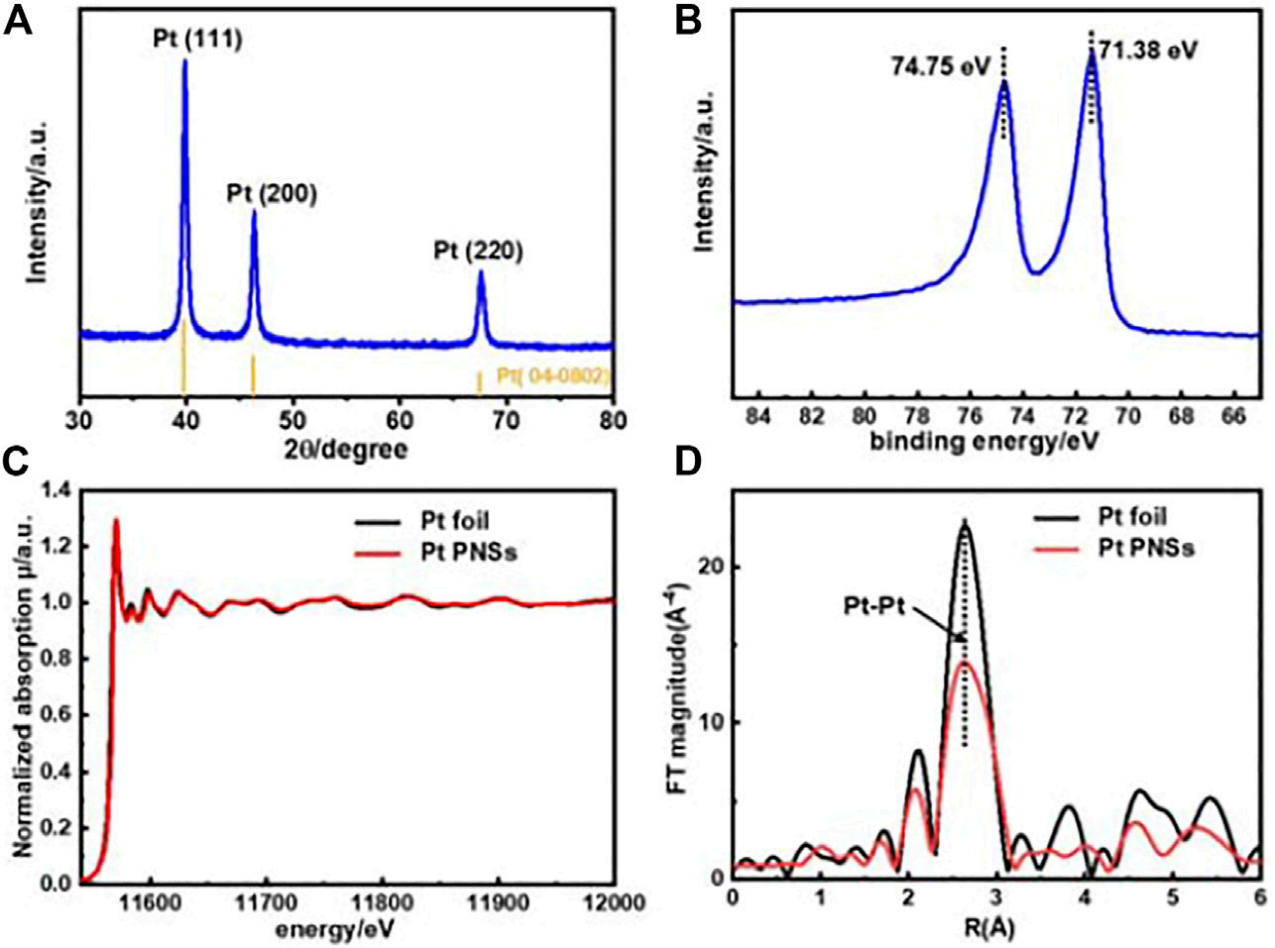
Spectroscopy characterizations of Pt PNSs. **(A)** XRD pattern, **(B)** XPS Pt4f spectrum, **(C)** XANES spectra, and **(D)** Fourier-transformed-extended X-ray absorption fine structure (FT-EXAFS) curves.

The composition and structure of the as-prepared Pt PNSs were further validated by XRD, XPS, and X-ray absorption near-edge structure (XANES) characterizations. As shown in Figure 2A, there are three diffraction peaks in the XRD pattern located at 39.76, 46.24, and 67.45, respectively, fitting well with fcc-Pt (JCPDS No. 04-0802). The XPS spectrum reveals that the Pt 4f_2/7_ binding energy sited at 71.38 eV, coincident well with that of pure Pt nanoparticles (Lai et al., 2019). No Pt (II/Ⅳ)-related peak was detected in both XPS (Figure 2B) and XANES spectroscopy analysis (Figures 2C, D), elaborating complete reduction from Pt (NH_3_)_4_^2+^ to Pt (Lu et al., 2015; Meng et al., 2017).

As discussed earlier, H_2_ was employed to reduce Pt (NH_3_)_4_^2+^ into metallic Pt. It is reasonable to conclude that there was also H_2_ adsorbed on the surface of the as-formed metallic Pt species, and the adsorbed H_2_ might be further activated since Pt is a well-documented hydrogenation and dehydrogenation catalyst (Yan et al., 2017). It is also well known that Pt is a highly efficient catalyst for the H_2_-O_2_ combination reaction (Michaelides and Hu, 2001). Thus, once the H_2_-adsorbed Pt is exposed to the air, the combination reaction would be initiated according to the following equation.

$$H_{2}\left(g\right)+12O_{2}\left(g\right)=H_{2}O\left(g\right)\Delta_{r}H_{m}^{\theta }=-241.8\mathrm{kJ}\mathrm{mo}l^{-1}$$ (1)

The burst of the reaction produced great heat, which further triggered the burning of the rGO. The oxidation of rGO is also an intense exothermic reaction (Eq. 2).

$$C\left(s\right)+O_{2}\left(g\right)=CO_{2}\left(g\right)\Delta_{r}H_{m}^{\theta }=-393.5\mathrm{kJ}\mathrm{mo}l^{\mathrm{-1}}$$ (2)

**SCHEME 1:**
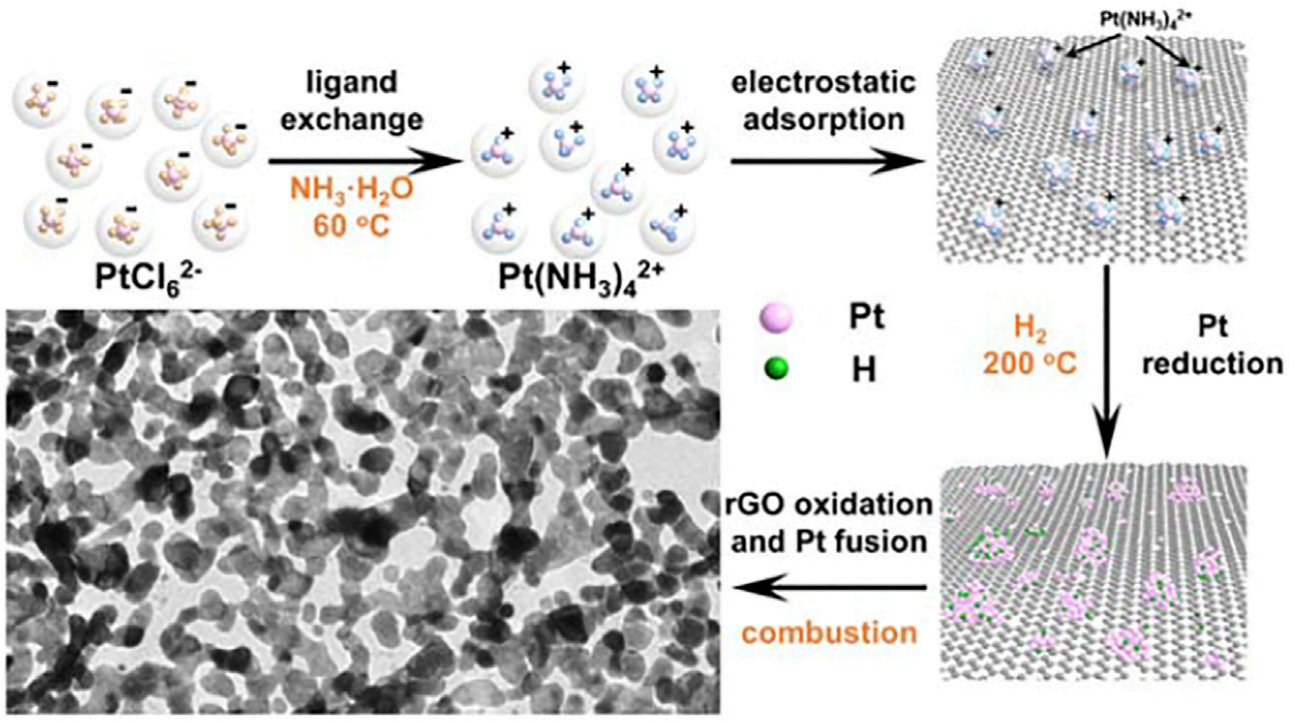
Schematic illustration of the synthesis process of Pt PNSs.

The released massive heat might induce the fusion of the adjacent Pt tiny species. Under the structural guidance of the rGO, 2D porous network structure was formed. Scheme 1 illustrates the whole process for the formation of the porous network structure.

To verify our speculation, we purged the system with N_2_ for another 2 h after the reduction of Pt (NH_3_)_4_^2+^ to remove the adsorbed H_2_ on metallic Pt (the products were denoted as Pt/rGO thereafter). Burning could not occur when exposing the N_2_-purged Pt/rGO to air. Thus, it could be confirmed that it was the exothermic effect of the H_2_-O_2_ combination reaction that sparked the combustion of the rGO. Pt nanoparticles could be easily identified for the N_2_-purged Pt/rGO during HAADF-STEM observation (Supplementary Figure S4A), as also demonstrated by the apparent diffractions assigned to fcc-Pt in the corresponding XRD pattern (Supplementary Figure S5A). High-resolution HAADF-STEM image reveal that there also existed large quantities of single atoms and clusters at the blank area of nanoparticles (insert in Supplementary Figure S4B). The coexistence of single atoms/clusters and nanoparticles were confirmed by XPS characterization (Supplementary Figure S5B). The Pt 4f_2/7_ XPS spectrum could be deconvoluted into two sets of peaks, one centered at 71.88 eV and another at 73.18 eV. The former one could be assigned to Pt nanoparticles, while the latter one could be ascribed to single Pt atoms or clusters (Lai et al., 2019). It should be pointed that even the signal from Pt nanoparticles also deviated from the binding energy of metallic Pt (0), which might be aroused by the electron interaction among the nanoparticles and single atoms/clusters. It is reasonable to conclude that the Pt single atoms and clusters are highly active, which might be a key factor for the catalysis of the oxidation of the adsorbed H_2_ and the rGO combustion.

Pure rGO without introduction of the Pt precursor was also treated under the same condition. As expected, the rGO did not burn as exposed to air after annealing in H_2_/Ar at 200°C for 2 h. Even additional intense flame could not trigger the combustion of the rGO (Supplementary Figure S6). Contrastively, the combustion could be initiated for the N_2_-purged Pt/rGO (Supplementary Figure S7). The distinctly different phenomena suggested that the Pt species also catalyzed the oxidation of rGO.

The loading amount of Pt was also a key parameter for the successful construction of the porous nanosheets. When the introduced H_2_PtCl_6_ was of 2 µM, self-ignition could not be observed for the as-prepared Pt/rGO when exposed to air after the H_2_ annealing process. And the combustion could only occur within a very small range instead of transferring to the whole sample even with a flame to trigger the oxidation (Supplementary Figure S8). It is easy to accept that the lower loading amount of Pt on rGO would produce lower concentration of active sites. Thus, the rGO oxidation was only limited in the local area of the flame. The spontaneous combustion could not be observed for the as-prepared Pt/rGO until the amount of the added H_2_PtCl_6_ was raised up to 10 µM. However, the Pt nanoparticles instead of network-like structure dominated the products, and the residual rGO could be easily identified in this case (Supplementary Figure S9), which implied that the loading amount of Pt was still relatively low. Although the active site concentration was high enough to trigger the combustion of rGO, the larger interspecies distance was not sufficient for their interconnection and thus the formation of the network structure. Therefore, a quite high Pt loading is an essential factor for the successful construction of the network-like nanosheet structure.

**FIGURE 3:**
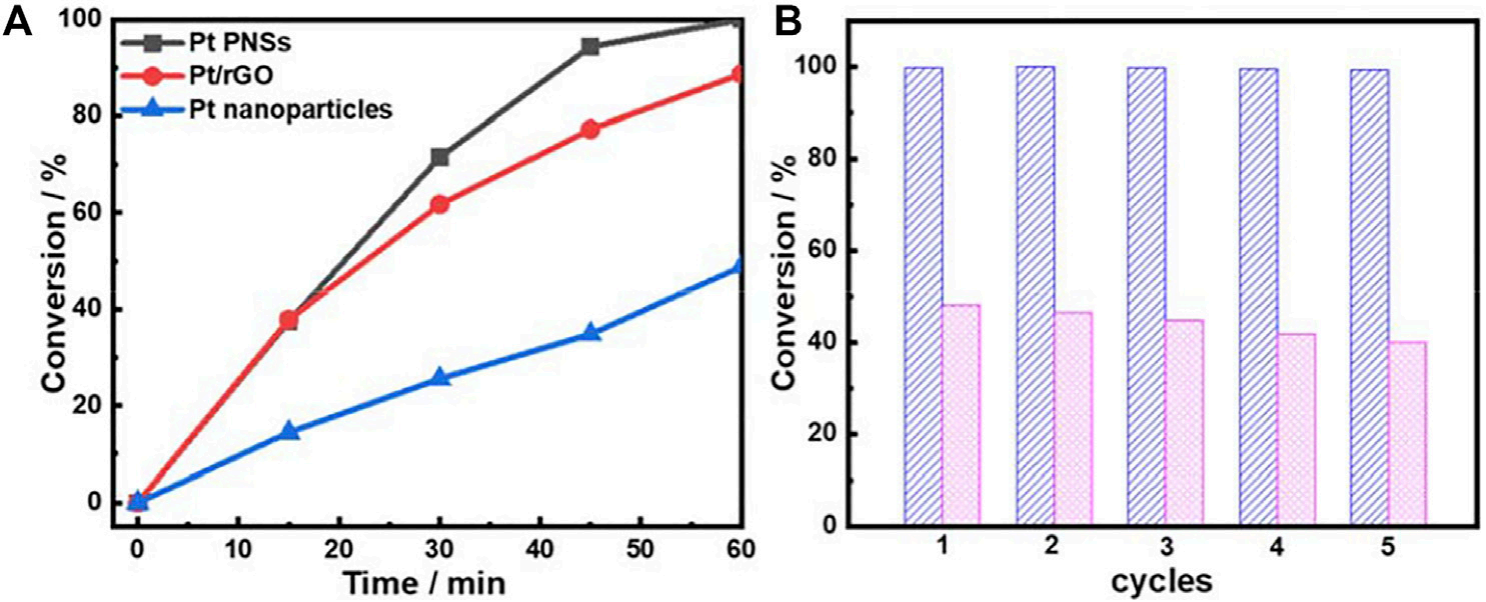
**(A)** conversions of styrene hydrogenation as a function of reaction time over the different catalysts at room temperature and **(B)** recycling data for (blue) Pt PNSs and (pink) Pt nanoparticles.

Inspired by the unique network-like nanosheet structure, we investigated the catalytic performance of the as-prepared Pt PNSs towards the styrene hydrogenation reaction, which is recognized as a model hydrogenation reaction. For comparison, N_2_-purged Pt/rGO hybrids and Pt dendritic structure with nanoparticles as building blocks were used as references. Supplementary Figure S10 demonstrated the diameter of the Pt nanoparticle building block was about 20 nm, similar to that of the segments in the Pt PNSs. However, the interconnection among the particle building blocks in the dendritic structures was far insufficient compared with the Pt PNSs. Figure 3A compares the conversions of styrene against reaction time. Pt PNSs presented an excellent hydrogenation performance with the completely conversion from styrene into phenylethane within 1 h. For the N_2_-purged Pt/rGO catalysts, although the conversion rate in the initial 15 min was similar to that of Pt PNSs, it suffered a subsequent deterioration. Only ∼88% of the styrene was converted after 1 h. Using Pt nanoparticles as the catalyst, the conversion rate was greatly reduced, and the conversion percent only reached 48% after 1 h under the same reaction conditions. The catalytic results showed that the Pt PNSs exhibited outstanding catalytic hydrogenation activity.

To further evaluate the intrinsic activity of the catalysts, the initial (in the first 0.5 h) turnover frequency (TOF) values were calculated. The TOF value of Pt PNSs was 158.14 h^−1^, which was nearly 3 times higher than that of Pt nanoparticles (TOF: 56.53 h^−1^). In recent years, intensive efforts have been devoted to introducing another transition metal into noble metal catalysts system to modulate the electronic structure by ligand and/or stain effects to optimize the catalytic performance. Our results indicate that the activity of the Pt PNSs is comparable to the reported unsupported bimetallic catalysts (Supplementary Table S1), even superior to some Pt-M bimetal system (M = Fe, Co, Ni, etc.) (Wang et al., 2014; Wang et al., 2015). We believe both the high surface area of the nanosheet structure and the interconnected network feature contribute to the superior activity. The former guaranteed the high concentration of active sites, and the latter accelerated the charge transfer during the catalyzation.

Recycling experiments were carried out to examine the catalytic durability of Pt PNSs and Pt nanoparticles catalysts (Figure 3B). Remarkably, conversion decline could be hardly identified over Pt PNSs after five recycles, whereas for Pt nanoparticles, the conversion decreases from 48% to 40% after five runs. The contrasting results demonstrated that Pt PNSs possessed much higher durability relative to the Pt nanoparticles. It is known that nanostructured materials often suffer from aggregations during the catalytic process, which is a predominant factor leading to the activity decline of nanocatalysts (Cheng et al., 2016). In our case, Pt PNSs could be well dispersed in the solvent by slightly shaking the container, and no precipitate was identified during the entire catalytic reaction process. In contrast, strong ultrasonication was necessary to get a better dispersion of Pt nanoparticles, and it always sedimented rapidly even under continuous stirring. Supplementary Figure S11 shows the clearly contrastive dispersion state of the nanosheets and the nanoparticles after the catalytic reaction. Obviously, the two-dimensional structure impeded the Pt PNSs from aggregations. The good dispersibility and excellent anti-aggregation performance resulted in the excellent durability of the Pt PNSs.

**FIGURE 4:**
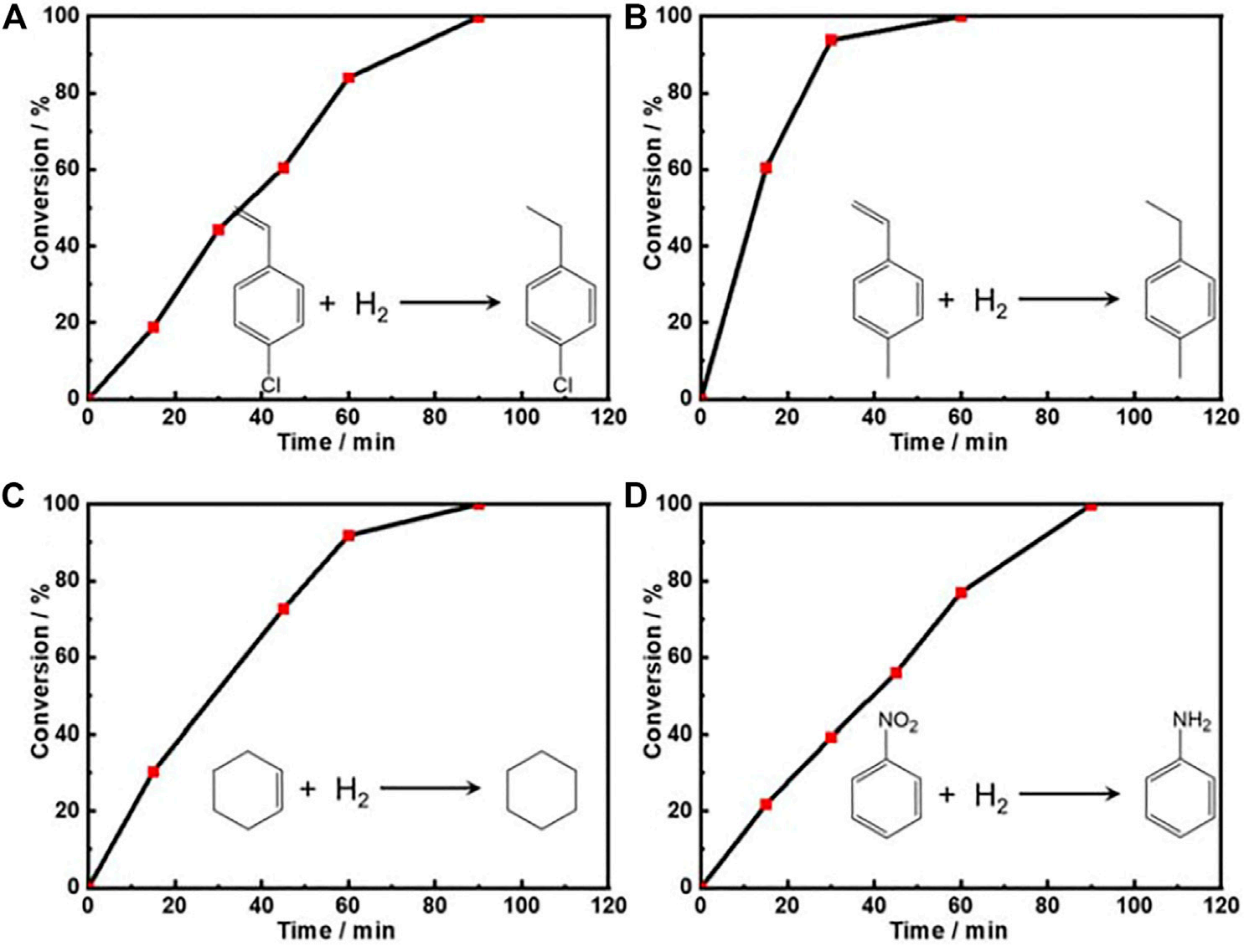
The plots of conversion versus time for the hydrogenation of **(A)** p-chlorostyrene, **(B)** p-methylstyrene, **(C)** cyclohexene, and **(D)** nitrobenzene over the Pt PNSs catalyst under room temperature and normal pressure with a H_2_ balloon as the H_2_ supplier.

Moreover, we also explored the catalytic hydrogenation performance of the as-prepared Pt PNSs towards other olefins (p-chlorostyrene, p-methylstyrene, and cyclohexene) and nitrobenzene. Figure 4 shows that the hydrogenation of all the four kinds of molecules could be completed within 90 min. Nitrobenzene was converted into aniline, and the hydrogenation of the olefins are on the non-aromatic unsaturated sites. The TOF values at 0.5 h were calculated as 90.47, 184.92, 136.17, and 86.63 h^−1^ for p-chlorostyrene, p-methylstyrene, cyclohexene, and nitrobenzene, respectively. The competitive data demonstrated the dramatic hydrogenation catalytic ability of Pt PNSs towards unsaturated bond.

## Conclusion

In summary, self-supported Pt porous nanosheets were successfully achieved by an *in situ* fusion strategy using rGO as both energy supplier and structure-directing templates. Tiny Pt species were firstly uniformly loaded onto rGO by an ion-adsorption and subsequent *in situ* H_2_ reduction process, which catalyzed the exothermic H_2_-O_2_ combination reaction as exposed to the air. The generated heat triggered the Pt-catalyzed rGO combustion, which in turn induced the *in situ* fusion of the Pt species into porous nanosheets under the 2D templating of rGO. The Pt PNSs exhibited great potential as hydrogenation catalyst by presenting outstanding catalytic hydrogenation activities towards nitrobenzene and different olefins such as styrene, p-chlorostyrene, p-methylstyrene, and cyclohexene. The styrene could be completely converted within 60 min under ambient conditions. The TOF value was as high as 158.14 h^−1^, three times higher than Pt nanoparticles. The satisfying performance was attributed to the enhanced surface area and the interconnected structure, which ensured both rich active sites and accelerated charge transfer rate. Furthermore, the network-like 2D structure gave the catalyst good dispersibility and aggregation resistance, which endowed the Pt PNSs with excellent recyclability.

## Data Availability

The raw data supporting the conclusion of this article will be made available by the authors, without undue reservation.
