# Supplementary material for: Network-Like Platinum Nanosheets Enabled by a Calorific-Effect-Induced-Fusion Strategy for Enhanced Catalytic Hydrogenation Performance
Source: Front Chem. 2022 Jan 5;9:818900. doi: 10.3389/fchem.2021.818900 (PMC8766668; doi:10.3389/fchem.2021.818900)
Supplement: Supplementary file 1 [file DataSheet1.docx]

Supplementary Material

Network-like platinum nanosheets enabled by a calorific-effect-induced-fusion strategy for enhanced catalytic hydrogenation performance

Tingwen Chen^1, 2†^, Dawei Pang^3†^, Jianxin Kang^1^, Dongfeng Zhang^1*^, Lin Guo^1*^

^1^School of Chemistry, Beijing Advanced Innovation Center for Biomedical Engineering, Key Laboratory of Bio-Inspired Smart Interfacial Science and Technology, Beihang University, Beijing 100191, China

^2^School of Physics, Beihang University, Beijing, 100191, China

^3^Institute of Microstructure and Property of Advanced Materials, Beijing University of Technology, Beijing, 100124, China

*** Correspondence:**Corresponding Author
dfzhang@buaa.edu.cn (D. F. Zhang); guolin@buaa.edu.cn (L. Guo)


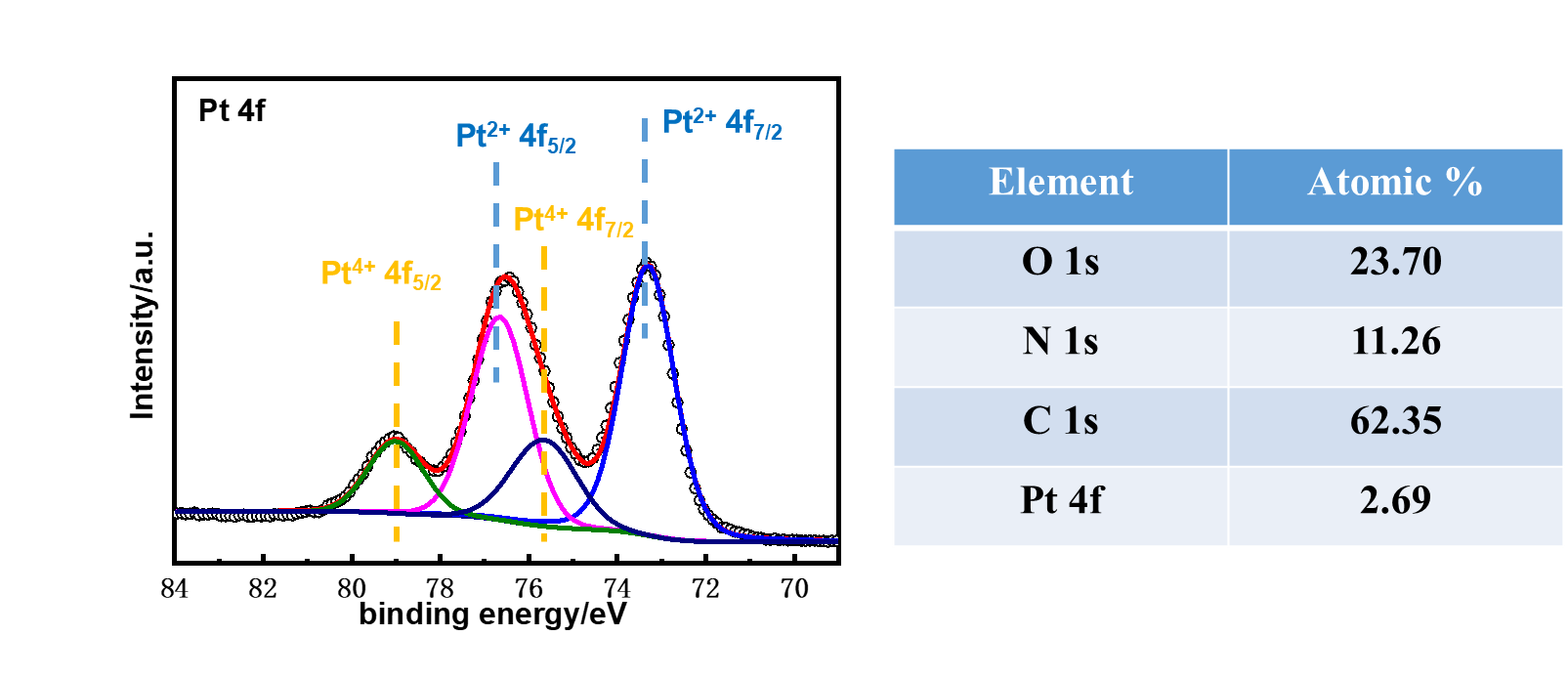


**Supplementary Figure 1.** Pt4f XPS spectra of Pt(NH_3_)_4_^2+^/rGO and the corresponding composition.


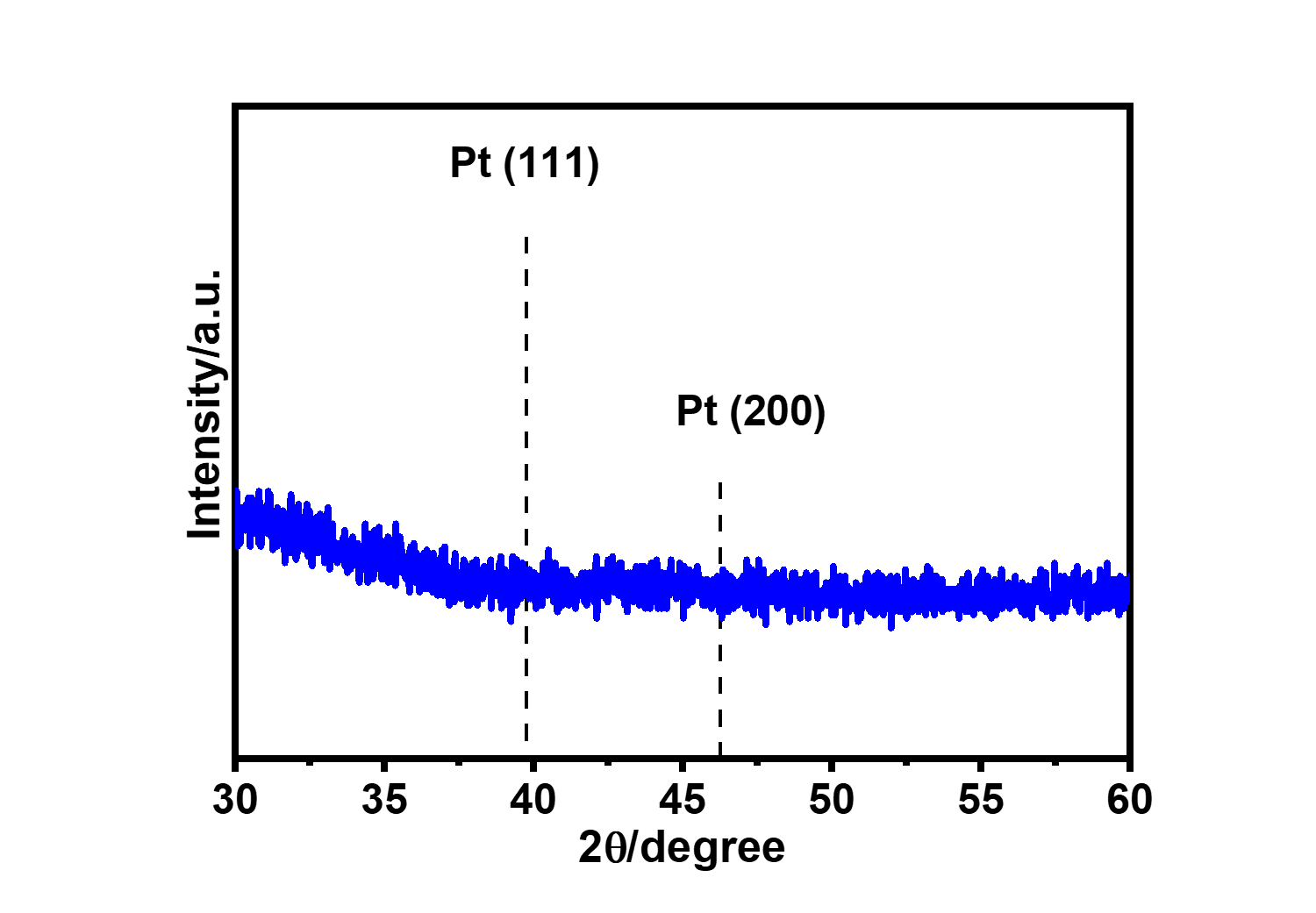


**Supplementary Figure 2.** XRD pattern of Pt(NH_3_)_4_^2+^/rGO.

**
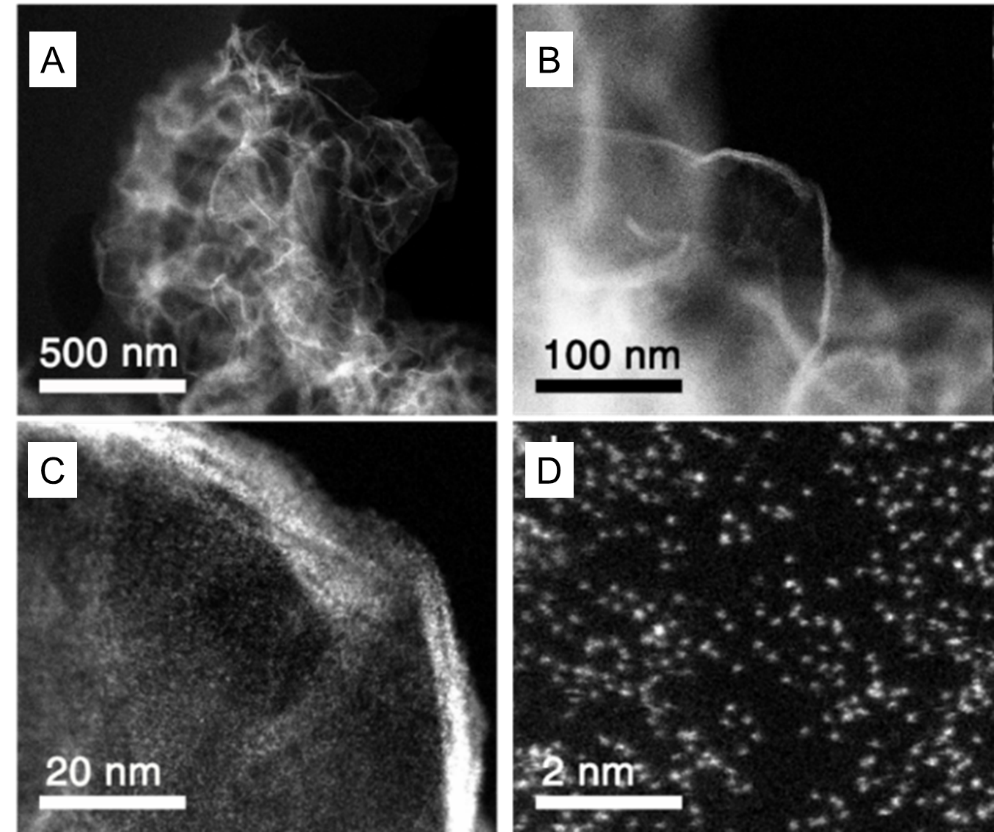
**

**Supplementary Figure 3.** The HAADF-STEM images of Pt(NH_3_)_4_^2+^/rGO with different magnifications.

**
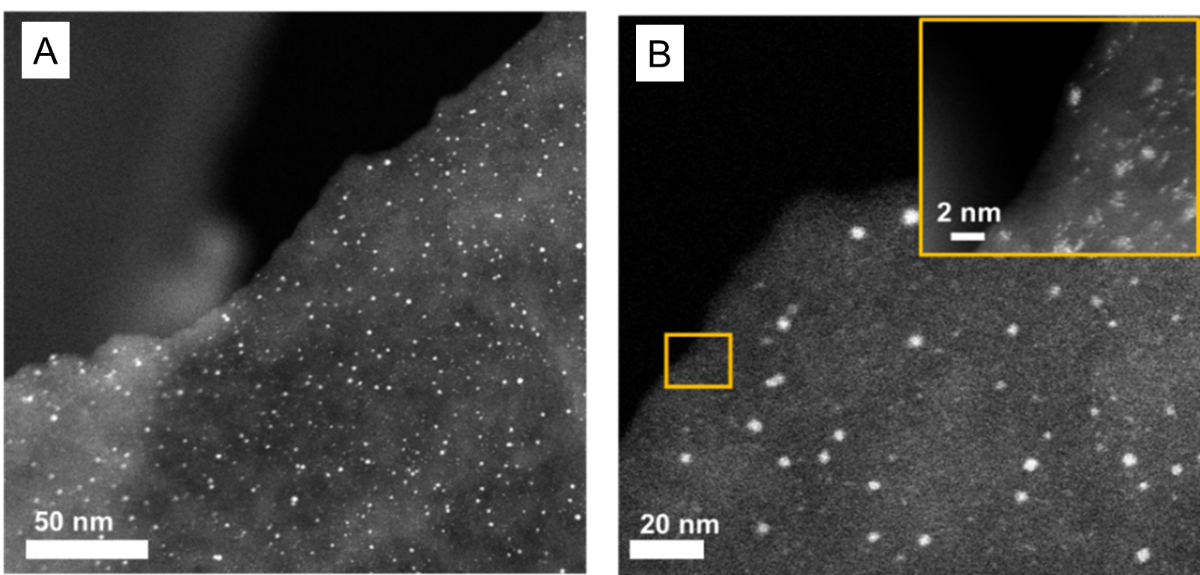
**

**Supplementary Figure 4.** The HAADF-STEM images of Pt/rGO with different magnifications.

**
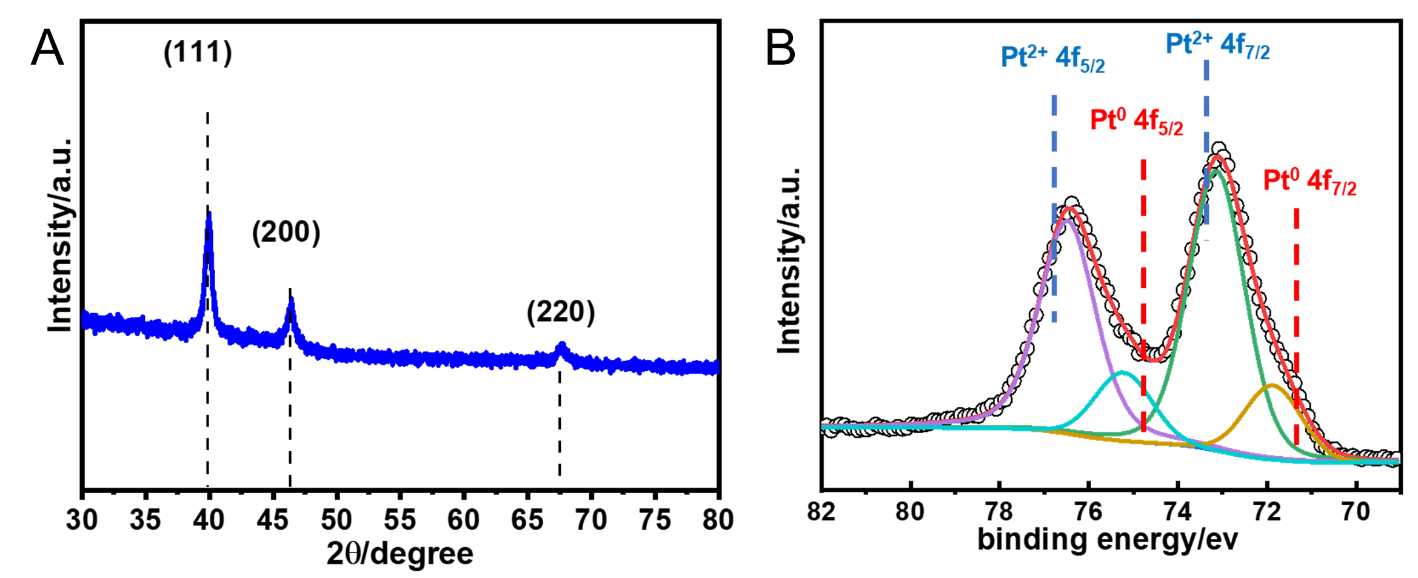
**

**Supplementary Figure 5.** (A) XRD pattern and (B) XPS Pt4f spectra of the as-prepared Pt/rGO..


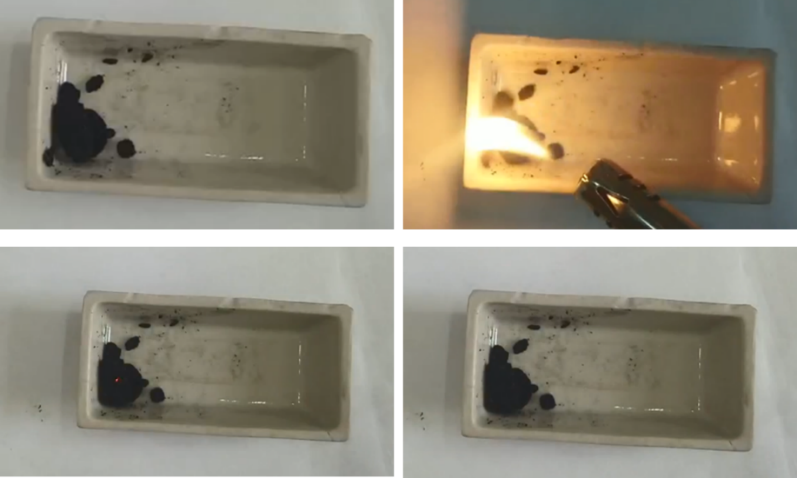


**Supplementary Figure 6.** Photographs show that burning not happened for the pure rGO even under intense flame.


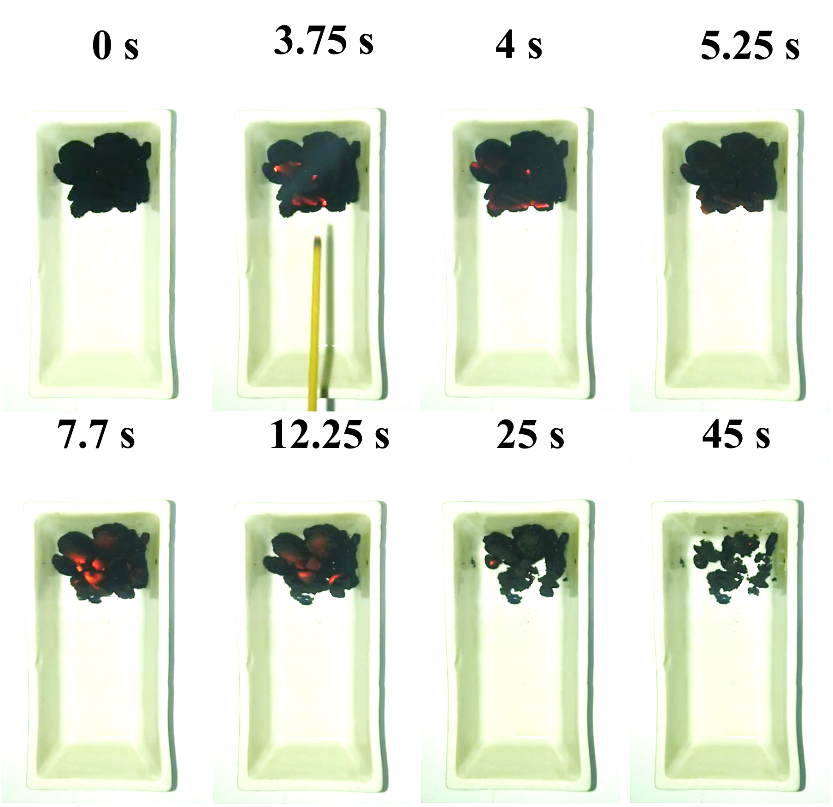


**Supplementary Figure 7.** Photographs show that even spark could trigger the combustion of the Pt/rGO formed with the feeded PtCl_6_^2-^ of 0.16 mM.

**
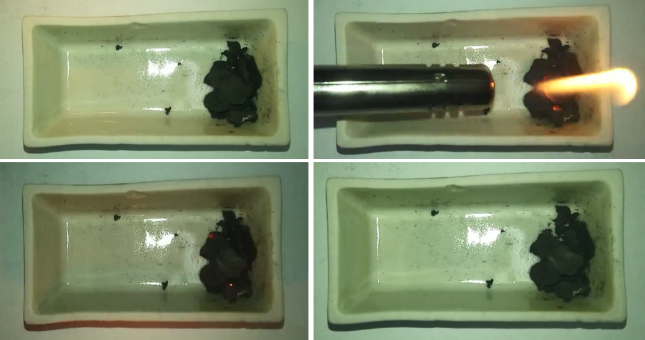
**

**Supplementary Figure 8.** Photographs show burning could be identified in a small region under intense flame for the Pt/rGO formed with the feeded PtCl_6_^2-^ of 2 uM.

**
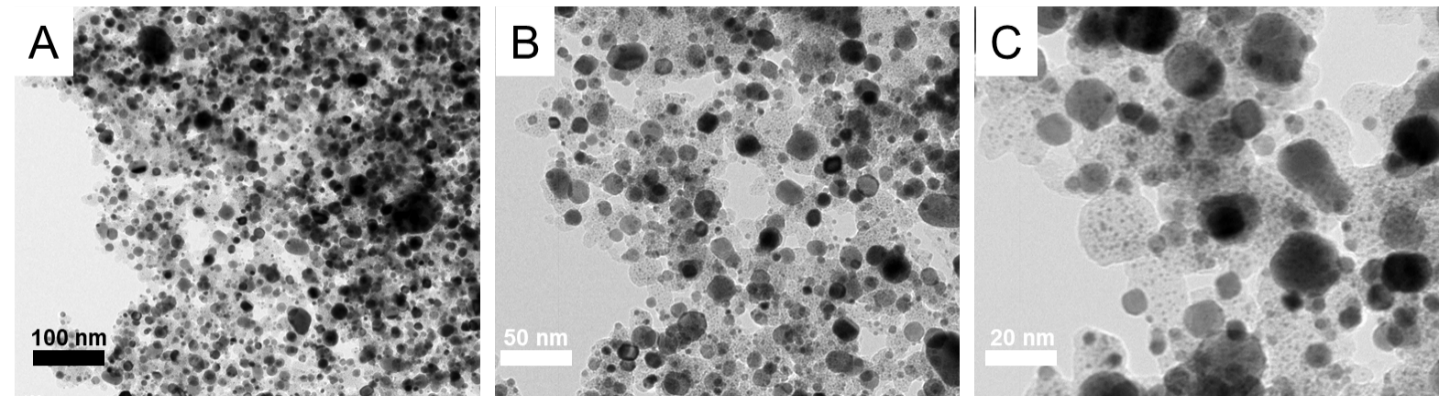
**

**Supplementary Figure 9.** The TEM images of the product collected after the combusting experiment of Pt/rGO with the feeded PtCl_6_^2-^ of 10 uM.





**Supplementary Figure 10.** The TEM image of Pt nanoparticles used in catalytic activity contrast test.


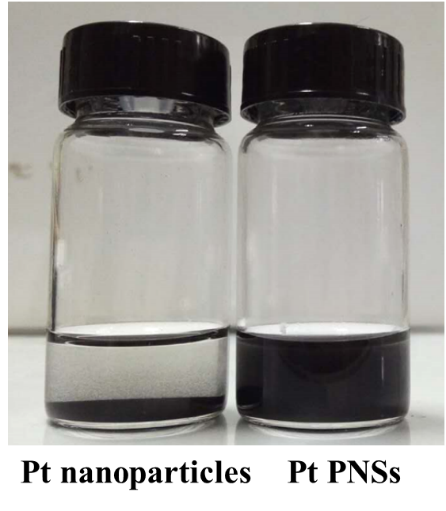


**Supplementary Figure 11.** The comparison of dispersibility of Pt nanoparticles and Pt PNSs after catalytic hydrogenation of styrene.

**Supplementary Table 1.** The catalytic hydrogenation of styrene by various reported catalysts

| **Catalyst** | **[Styrene]/[Pt]**  **(mol/mol)** | **Temperature** | **H_2_ pressure** | **TOF** | **Ref.** |
| --- | --- | --- | --- | --- | --- |
| **Pt_3_Fe concave nanocubes** | 14.2 | RT | 1 atm | 30.38 h^-1^ | Wang *et al.*, 2015 |
| **Pd/MOF-5** | 158 | 35 ^o^C | 1 atm | 17.6 h^-1^ | Sabo *et al.*, 2007 |
| **Pt_3_Co concave nanocubes** | 16 | RT | 1 atm | 30.74 h^-1^ | Wang *et al.*, 2014 |
| **Pt_42_Sn_58_ Wavy Nanowires** | 47 | NA | 1 bar | 70.78 h^-1^ | Ding *et al.*, 2015 |
| **Pt PNSs** | **111** | **25℃** | **1 atm** | **158.14 h^-1^** | **This work** |
| **Pt nanoparticles** | **111** | **25℃** | **1 atm** | **56.53 h^-1^** | **This work** |

**References:**

Ding, J., Bu, L., Zhang, N., Yao, J., Huang, Y. and Huang, X. (2015). Facile synthesis of ultrathin bimetallic PtSn wavy nanowires by nanoparticle attachment as enhanced hydrogenation catalysts,  *Chem. Eur. J.* 21, 3901-3905. doi: 10.1002/chem.201406069

Sabo, M., Henschel, A., Froede, H., Klemm, E. and Kaskel, S. (2007). Solution infiltration of palladium into MOF-5: synthesis, physisorption and catalytic properties,  *J. Mater. Chem.* 17, 3827-3832. doi: 10.1039/b706432b

Wang, C., Lin, C., Zhang, L., Quan, Z., Sun, K., Zhao, B., *et al.* (2014). Pt_3_Co concave nanocubes: synthesis, formation understanding, and enhanced catalytic activity toward hydrogenation of styrene,  *Chem. Eur. J.* 20, 1753-1759. doi: 10.1002/chem.201301724

Wang, C., Lin, C., Zhao, B., Zhang, L., Kumbhar, A., Fan, G., *et al.* (2015). High-indexed Pt_3_Fe nanocatalysts and their enhanced catalytic performance in dual organic reactions, *Chemnanomat* 1, 331-337. doi: 10.1002/cnma.201500048
